# Renal Implications of Dysregulated Protein Homeostasis: Insights into Ubiquitin–Proteasome and Autophagy Systems

**DOI:** 10.3390/biom15030349

**Published:** 2025-02-28

**Authors:** Charlotte Delrue, Marijn M. Speeckaert

**Affiliations:** 1 Department of Nephrology, Ghent University Hospital, 9000 Ghent, Belgium; charlotte.delrue@ugent.be; 2 Research Foundation-Flanders (FWO), 1000 Brussels, Belgium

**Keywords:** ubiquitin–proteasome system, autophagy, chronic kidney disease, protein homeostasis

## Abstract

The ubiquitin–proteasome system (UPS) and autophagy maintain protein homeostasis, which is critical to cellular function and survival. The dysregulation of these pathways has been recognized as a hallmark of acute kidney injury and chronic kidney disease. This review elucidates the role of the UPS and autophagy in kidney disease, namely through inflammation, oxidative stress, fibrosis and apoptosis. The pathways of NF-κB, TGF-β and mitochondrial failure result in glomerular injury and tubulointerstitial fibrosis due to impaired proteostasis in podocytes and tubular epithelial cells. Recent studies have revealed a connection between the autophagic process and the UPS, wherein compensatory mechanisms aim to spike down proteotoxic stress but eventually seem inadequate in cases of chronic derangement. Low-dose pharmacological inhibitors, autophagy modulators, and new gene and nanotechnology-based treatments may all help to restore the protein balance and reduce kidney injury. A more thorough understanding of these pathways is needed to develop kidney-protective and disease-modifying therapeutic interventions.

## 1. Introduction

Protein homeostasis (proteostasis) is the delicate balance of protein synthesis, folding, trafficking, and degradation essential for normal cellular function. Protein degradation in cells is primarily performed by the ubiquitin–proteasome system (UPS) and autophagy. The UPS targets short-lived or misfolded soluble proteins, while autophagy clears the long-lived proteins, insoluble aggregates, and even organelles. Together, these mechanisms promote cellular and tissue homeostasis under physiological and stressed conditions [1].

The UPS and the process of autophagy are increasingly recognized as integral to cellular quality control. Their dysregulation is associated with myriad diseases, which include neurodegenerative disorders, cancer, acute kidney injury (AKI) and chronic kidney disease (CKD) [2]. In the kidneys, protein turnover is critical for the functions of nephrons as well as glomerular maintenance. The UPS and autophagy control processes that drive inflammation, fibrosis, and oxidative stress [3]. Impaired proteasomal degradation in podocytes contributes to CKD, possibly due to increased apoptosis, accumulation of ubiquitinated proteins, and oxidative stress [4]. In a similar vein, impaired autophagy in renal cells drives fibrosis and mitochondrial dysfunction, underscoring the importance of both systems for kidney health [5].

This review looks at the function of dysregulated protein homeostasis in kidney diseases, with a focus on the UPS–autophagy interaction. It examines the molecular mechanisms that cause their malfunction, their role in kidney disease development, and prospective treatment approaches to restore proteostasis.

## 2. Dysregulated Protein Homeostasis in Kidney Disease

The intricate interplay between inflammation, oxidative stress, and fibrosis plays a crucial role in the progression of kidney disease. Therefore, maintaining effective control over the unfolded protein response (UPR) and autophagy is essential to prevent the worsening of this trio, resulting in severe pathogenic effects [4,5]. In terms of kidney injury, disrupted protein homeostasis can stimulate inflammatory pathways through various interconnected mechanisms. The UPR is a stress-induced signaling system that restores the protein balance and is activated when misfolded proteins accumulate in the endoplasmic reticulum (ER). However, if ER stress persists, the UPR switches from a protective to a pathogenic mechanism. Aggregated proteins activate toll-like receptors (TLRs), particularly TLR4, which recognize them as damage-associated molecular patterns [6]. The process described activates pro-inflammatory pathways through the inositol-requiring enzyme 1 (IRE1), protein kinase RNA-like endoplasmic reticulum kinase (PERK) and activating transcription factor 6 (ATF6) signaling pathways. This results in an increase in the production of pro-inflammatory cytokines such as tumor necrosis factor-alpha (TNF-α), interleukin (IL)-6, IL-1β, and monocyte chemoattractant protein (MCP)-1. The pathways that are triggered lead to an intensified burst of cytokine production, which contributes to inflammation [7,8]. In diabetic nephropathy (DN), TNF-α can enhance podocyte damage by activating the nuclear factor kappa-light-chain-enhancer of activated B cell (NF-κB) signaling and leading to tubular epithelial cell dysfunction, thereby accelerating disease progression. Additionally, these cytokines can attract and activate immune cells like T cells and macrophages, which infiltrate the renal glomeruli and interstitium, ultimately causing chronic inflammation. DN can be exacerbated by TNF-α when it prompts NF-κB signaling and triggers podocyte damage and tubular epithelial cell dysfunction. Eventually, this accelerates disease progression. Furthermore, TNF-α can draw in immune cells, including T cells and macrophages, that penetrate the renal glomeruli and interstitium, leading to chronic inflammation [9].

Proteostasis failure is a condition that worsens oxidative stress by changing mitochondrial activity, leading to increased reactive oxygen species (ROS) formation. This, in turn, triggers fibrosis and structural damage in the kidneys through the activation of the NF-κB pathway [9]. Misfolded or aggregated proteins reduce mitochondrial respiration’s effectiveness, thereby reducing ATP generation and increasing electron leakage. The leaked electrons increase the production of ROS, which further harm mitochondrial proteins, lipids, and DNA, exacerbating mitochondrial dysfunction. Furthermore, poor autophagy, or mitophagy, slows the clearance of damaged mitochondria, worsening ROS buildup and extending oxidative stress [10]. The oxidation induced by ROS causes changes in biological components, including membrane phospholipids, leading to lipid peroxidation and oxidized proteins that lose their function and tend to aggregate. These processes affect key cellular processes such as calcium signaling and energy utilization, which worsen mitochondrial dysfunction. This ultimately leads to impaired cellular function [11]. Renal tubular epithelial cells (TECs) have high levels of ROS due to impaired proteasomal and autophagic activity, which worsens tubular injury by activating proinflammatory and profibrotic pathways. This leads to an increase in the production of transforming growth factor-beta (TGF-β), a crucial element in renal fibrosis, and eventually leads to the epithelial-to-mesenchymal transition (EMT) and deposition of extracellular matrix (ECM) in the kidneys [12]. In CKD, the activation of nuclear factor erythroid 2-related factor 2 (Nrf2) by ROS serves as a protective mechanism that enhances antioxidant defenses. Unfortunately, due to the persistent nature of oxidative stress in CKD, this system eventually becomes overwhelmed and unable to provide sufficient protection [13].

In the development of CKD, renal fibrosis is a hallmark and is heavily influenced by dysregulated protein homeostasis. The complexity of the problem lies in the accumulation of insoluble aggregates and misfolded proteins, which instigates cellular stress responses. These responses may include oxidative stress and inflammation, which lead to the activation of fibrogenic pathways. Primarily, the TGF-β signaling cascade is activated during these events. TGF-β signaling is responsible for converting fibroblasts to myofibroblasts, which is the primary cause of fibrosis. Myofibroblasts promote excessive production of ECM proteins like fibronectin, laminin, collagen I, and collagen IV, which significantly contribute to tubulointerstitial fibrosis and glomerulosclerosis [14]. The normal turnover of ECM proteins is disrupted when proteostasis is impaired, leading to abnormal accumulation in the renal interstitium. Proteostasis mechanisms, such as the UPS and autophagy, are responsible for breaking down and recycling ECM components. If these systems are disrupted, an excessive amount of ECM proteins accumulates, causing the interstitial matrix to stiffen and impede tissue repair. This indicates that proteostasis is crucial to maintaining the balance of ECM protein turnover in the kidney [15]. The presence of modified ECM proteins, such as advanced glycation end-products (AGEs), in DN intensifies the renal impairment by increasing the levels of oxidative stress and inflammation. Additionally, the damage to kidney function is exacerbated due to these modifications [16]. To contribute to fibrosis, ongoing inflammation releases pro-inflammatory cytokines such as IL-6 and TNF-α. These cytokines enhance TGF-β signaling and activate myofibroblasts. Mitochondrial malfunction and ROS generation cause oxidative stress, exacerbating ECM deposition and cellular death. This ROS-induced damage also triggers the activation of secondary fibrogenic mediators like connective tissue growth factor (CTGF), which further exacerbates fibrosis [17]. The deterioration of kidney function in severe CKD is aggravated by the interplay between inflammation, oxidative stress, and ECM buildup. The rigidity of the fibrotic matrix hampers the diffusion of oxygen and nutrients, leading to hypoxia and escalated TGF-β activity. This sets off a cycle of self-propagating fibrosis and renal harm, causing the irreparable decline of renal function that characterizes advancing CKD [18]. The accumulation of fibrosis can be attributed to the increased nicotinamide adenine dinucleotide phosphate (NADPH) oxidase and TGF-β1 activity caused by oxidative stress. This results in heightened ECM synthesis and decreased matrix turnover [12]. Circular RNAs, such as circACTA2, have been identified as important regulators of fibrotic pathways in DN and have the potential to serve as both biomarkers and therapeutic targets for renal fibrosis. These non-coding RNAs play a role in fibrotic signaling by acting as “sponges” for microRNAs that target genes related to the TGF-β ECM [19]. An effective treatment for fibrosis involves inhibiting TGF-β signaling and improving autophagic flux through proteostasis treatment. Pirfenidone and Nrf2 activators are promising drugs currently being researched to manage fibrosis by reducing the trio of inflammation, oxidative stress, and excessive ECM components [20,21].

**Figure 1:**
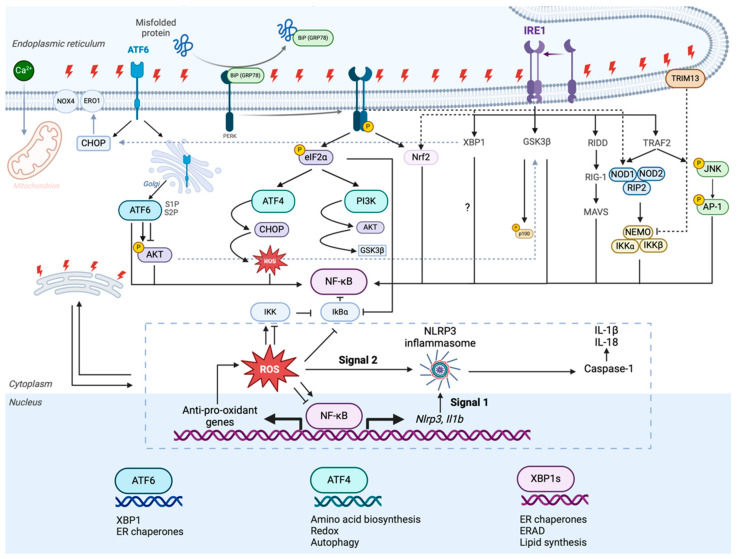
The unfolded protein response (UPR) pathways and their interconnection with inflammatory signaling. The UPR is initiated in response to the accumulation of misfolded proteins in the endoplasmic reticulum (ER) and involves three primary stress sensors: activating transcription factor 6 (ATF6), protein kinase RNA-like endoplasmic reticulum kinase (PERK), and inositol-requiring enzyme 1 (IRE1). Each of these sensors activates distinct signaling pathways that contribute to restoring ER homeostasis or triggering inflammation and apoptosis when the stress is unresolved. Upon ER stress, binding immunoglobulin protein [BiP, 78 kDa glucose-regulated protein (GRP78)] dissociates from ATF6, enabling ATF6 to translocate to the Golgi apparatus, where it is cleaved by site-1 protease (S1P) and S2P protease. The cleaved, active ATF6 fragment enters the nucleus and promotes the expression of ER chaperones [e.g., X-box binding protein 1 (XBP1)]. Additionally, ATF6 signaling can activate AKT (protein kinase B), which, in turn, contributes to the regulation of the nuclear factor kappa-light-chain-enhancer of activated B cells (NF-κB) pathway by phosphorylating IKK (IκB kinase). This results in the phosphorylation and degradation of IκBα, releasing NF-κB to translocate into the nucleus, where it regulates the expression of inflammatory and survival genes. Furthermore, under ER stress, PERK becomes active upon BiP dissociation and phosphorylates eukaryotic initiation factor 2α (eIF2α), which reduces global protein synthesis to alleviate the ER burden. However, selective translation of ATF4 occurs, leading to the expression of genes related to amino acid biosynthesis, redox homeostasis, and autophagy. ATF4 also upregulates C/EBP homologous protein (CHOP), which can induce apoptosis. ATF4 signaling generates ROS, which play a dual role by enhancing oxidative stress and activating inflammatory responses. When BiP dissociates from IRE1, IRE1 becomes active and initiates the unconventional splicing of XBP1 mRNA, producing XBP1s, a transcription factor that promotes the expression of genes involved in ER chaperone production, ER-associated degradation (ERAD), and lipid synthesis. Additionally, IRE1 interacts with tumor necrosis factor receptor-associated factor 2 (TRAF2), which activates the IKK complex and the c-Jun N-terminal kinase pathway (JNK) pathway. JNK signaling leads to the activation of the transcription factor activator protein 1 (AP-1), which promotes the expression of pro-inflammatory genes. TRAF2 also activates receptor-interacting protein kinase 2 (RIP2), which in turn activates the nucleotide-binding oligomerization domain-containing protein 1 (NOD1) and NOD2 pathways, further contributing to NF-κB activation. Additionally, IRE1 signaling activates regulated IRE1-dependent decay (RIDD), which can influence inflammatory pathways through the degradation of specific mRNAs. The integration of UPR signaling with inflammatory pathways is facilitated by ROS production, which occurs downstream of PERK and IRE1 activation. ROS production provides a secondary signal necessary for the activation of the nucleotide-binding oligomerization domain- (NOD), leucine-rich repeat- (LRR), and pyrin domain-containing (PRC) protein 3 (NLRP3) inflammasome. The inflammasome activation requires signal 1, which is provided by NF-κB activation, resulting in the expression of Nlrp3 and interleukin-1 beta (IL-1b) genes. Activation of the NLRP3 inflammasome leads to the cleavage of pro-caspase-1 into active caspase-1, which processes pro-IL-1β and pro-IL-18 into their active, secreted forms (IL-1β and IL-18), driving inflammation.

The dysregulation of protein homeostasis has a substantial downstream impact on apoptosis, particularly in TECs, where continued ER stress triggers pro-apoptotic pathways. The ER stress-induced apoptosis is instigated by the escalation of C/EBP homologous protein (CHOP), which triggers the intrinsic apoptotic pathway through the activation of caspases and the release of mitochondrial cytochrome c, subsequently leading to tubular atrophy—a common characteristic of both AKI and CKD [22]. In order to reduce tubular cell death and improve renal outcomes in both AKI and CKD, targeting ferroptosis with ferrostatin-1 has been studied [23,24]. Figure 1 gives an overview of the different UPR pathways and their interconnection with inflammatory signaling and ROS.

## 3. The Ubiquitin–Proteasome System in Kidney Disease

The UPS is the primary non-lysosomal mechanism for controlled intracellular ATP-dependent proteolysis (Table 1). This process involves labeling proteins with ubiquitin molecules cascading via three enzymes, namely E1, E2, and E3, to provide specificity to the substrate. Polyubiquitin-tagged proteins are then identified and degraded by the 26S proteasome, which is a complex composed of multiple enzymes. This approach is critical to removing damaged, misfolded, or excess proteins in order to maintain cellular homeostasis [25,26,27]. In addition to destruction, ubiquitination regulates transcription, endocytosis, signal transmission, and other physiological activities [28].

In kidney diseases, the proper functioning of the UPS is crucial as it prevents protein aggregation and cellular stress. The UPS is responsible for removing misfolded, oxidized, and short-lived proteins. However, reduced UPS activity in kidney cells, particularly in high-demand cell types like podocytes and proximal TECs, worsens proteotoxic stress. When the proteasome malfunctions, polyubiquitinated proteins accumulate, leading to the formation of toxic aggregates that disturb cellular homeostasis and activate inflammation-promoting pathways. UPS dysfunction disproportionately affects podocytes, which are essential for maintaining the glomerular filtration barrier (GFB). Impairment of proteasome activity leads to an abnormal buildup of ubiquitinated cytoskeletal proteins, leading to proteinuria through the disruption of foot processes. Injured podocytes often exhibit overexpression of the deubiquitinating enzyme ubiquitin C-terminal hydrolase L1 (UCH-L1), causing impaired proteasomal degradation and exacerbating glomerular damage in conditions like membranous nephropathy and focal segmental glomerulosclerosis (FSGS) [36]. The malfunction of UPS in proximal TECs can lead to a decrease in the removal of misfolded proteins and mitochondria, resulting in oxidative stress and inflammation. This imbalance is particularly damaging in circumstances such as ischemic AKI, in which the need for protein degradation surpasses the proteasome’s capacity, resulting in cell death and kidney failure [37].

### 3.1. The Role of the Ubiquitin–Proteasome System in Acute Kidney Injury

**Figure 2:**
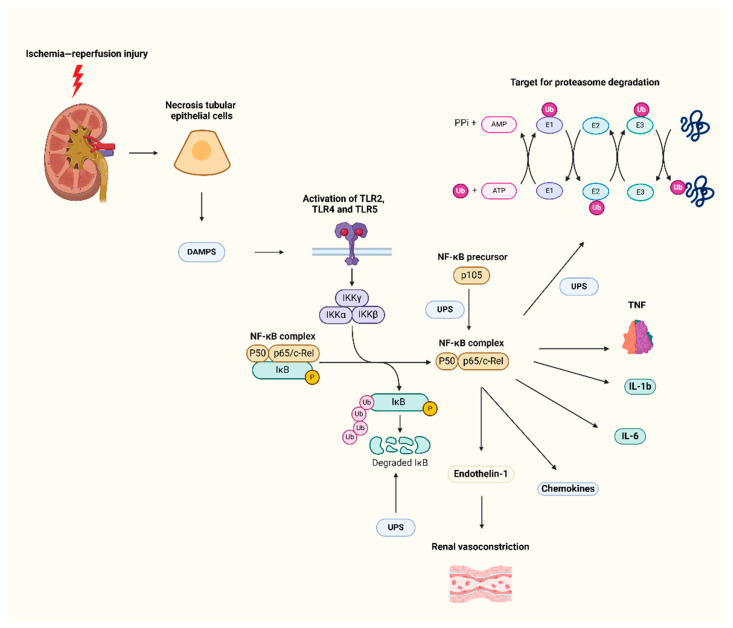
The role of UPS-mediated NF-κB activation in the pathogenesis of acute kidney injury. The ubiquitin–proteasome system (UPS) activates NF-κB during ischemia-reperfusion damage (IRI), resulting in acute kidney injury (AKI). Necrotic tubular epithelial cells produce damage-associated molecular patterns (DAMPs), which activate Toll-like receptors (TLR2, TLR4, and TLR5). Activating these receptors activates the IKK complex (IKKα, IKKβ, and IKKγ), which phosphorylates and destroys the nuclear factor kappa-light-chain-enhancer of activated B cells (NF-κB) inhibitor IκB through UPS. The NF-κB complex (p50/p65) is released and moves to the nucleus, triggering the production of pro-inflammatory cytokines [tumor necrosis factor (TNF), interleukin (IL)-1β, IL-6], chemokines, and endothelin-1. Endothelin-1 increases kidney damage by generating vasoconstriction. Additionally, the UPS converts the NF-κB precursor protein (p105) to the active p50 subunit. UPS regulates NF-κB activity by degradation via E3 ligases, highlighting its dual role as a positive and negative regulator.

In AKI, the UPS has two roles in cellular stress regulation. During AKI, the increasing oxidative stress and ischemia place an extra burden on the UPS’s ability to digest damaged and misfolded proteins. However, the increased burden may exceed the proteasome’s capacity, causing proteotoxic stress and necroinflammatory responses. Ischemic injury in TECs causes the buildup of polyubiquitinated proteins, activating inflammatory signaling pathways like NF-κB (Figure 2) [36]. This mechanism leads to cell damage and apoptosis. In AKI models, blocking specific proteasome subunits has been shown to diminish inflammatory cytokine production and tubular damage [38]. Proteasome dysfunction in AKI has an impact on mitochondrial dynamics, since defective degradation of mitofusin proteins causes mitochondrial fragmentation and ROS overproduction. This exacerbates oxidative stress and increases TEC death, accelerating the damage cycle [11].

In AKI models, inhibiting specific proteasome subunits reduces inflammatory cytokine production and tubular damage. Targeting the NF-κB pathway can reduce inflammation in AKI. The IKK complex phosphorylates IκB, causing NF-κB activation. Inhibitors of IKK can disrupt this mechanism and impede the production of inflammatory mediators. BAY 11-7082 inhibits NF-κB activity, decreasing renal inflammation in experimental animals. However, it is important to highlight that BAY 11-7082 may have off-target effects, and its specificity for IKK inhibition is unknown [39]. Proteasome inhibitors slow IκB breakdown, inhibiting NF-κB and reducing inflammation [40].

### 3.2. The Role of the Ubiquitin–Proteasome System in Chronic Kidney Disease

**Figure 3:**
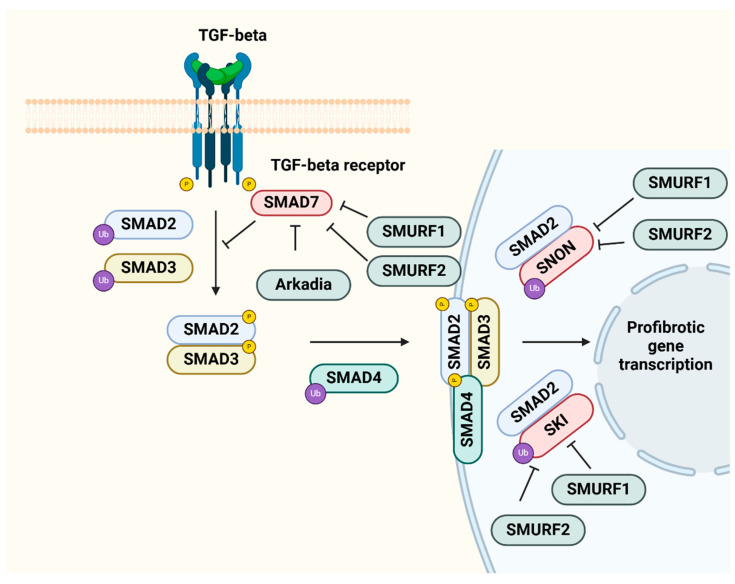
UPS modulation of TGF-β signaling in renal fibrosis. Illustration of the TGF-β signaling cascade and its control by the ubiquitin–proteasome system (UPS) in renal fibrosis. TGF-β binds to its receptor complex, which includes TGF-β receptor types 1 and 2, activating SMAD2 and SMAD3 by phosphorylation. Once activated SMAD2/3 binds to SMAD4, complexes translocate into the nucleus, inducing profibrotic gene expression. Besides binding to SMAD4, SMAD2/3 may also interact with transcriptional co-repressors like SNOM and SKI. These are complexes not conferring transcriptional activation. SMAD7 is a inhibitory SMAD that binds and inactivates the active TGF-β receptor, preventing SMAD2/3 activation. The ubiquitin-proteasome system (UPS) targets and breaks down TGF-β pathway components. E3 ubiquitin ligases, including SMURF1 and SMURF2, cause polyubiquitylation and degradation of SMAD2, SMAD3, SMAD4, and inhibitory regulators such as SNON and SMAD7, influencing pro- or anti-fibrotic signaling. Arkadia, an E3 ligase, degrades SMAD7 and increases TGF-β signaling. These interactions highlight the UPS’s dynamic regulation of TGF-β-mediated fibrogenesis, which contributes to renal fibrosis progression via extracellular matrix formation.

When it comes to CKD, persistent dysregulation of the UPS can contribute to long-term kidney damage. Renal cells can accumulate misfolded proteins and aggregates that are marked with ubiquitin, thereby activating fibrogenic pathways that involve signaling by TGF-β, which triggers the activation of myofibroblasts that deposit extracellular matrix (Figure 3) [36].

Additionally, this dysfunction in proteostasis can induce ER stress and the subsequent activation of the UPR, which ultimately exacerbates both the fibrotic and inflammatory processes [36]. The UPS is a crucial factor in regulating turnover of Nrf2, which is a transcription factor that protects against oxidative stress by acting as a master antioxidant. In CKD, excessive proteasomal degradation of Nrf2 reduces its ability to provide cytoprotection, which exacerbates oxidative stress and inflammation. Research on animal models has illustrated that targeted proteasome inhibition could potentially stabilize Nrf2, leading to the enhancement of antioxidant defenses, a reduction in ROS levels, and a decrease in renal fibrosis. Various compounds, such as bardoxolone methyl and sulforaphane, have been proven to elevate Nrf2 activity by modifying the UPS, and these have displayed positive results in improving renoprotection in CKD experimental models [41]. In addition to Nrf2, the UPS interacts with a variety of signaling pathways that influence the course of CKD. A dysfunctional UPS can alter Wnt/β-catenin signaling, leading to EMT and renal fibrosis. Modulating UPS activity can restore normal β-catenin turnover, potentially preventing fibrosis and maintaining kidney function [42].

The TGF-β signaling system plays a crucial role in renal fibrosis, promoting ECM deposition and myofibroblast activation. Therapeutic medications such as Pirfenidone and Nintedanib can inhibit TGF-β signaling, reduce inflammation, and prevent ECM buildup. These drugs target crucial fibrosis drivers, which makes them helpful in reducing disease progression [43]. Rapamycin and metformin are autophagy modulators that block mTOR or launch AMP-activated protein kinase (AMPK) with a focus on cellular homeostasis and minimization of fibrotic damage. Emerging techniques, such as gene therapy targeting autophagy-related genes, including *Beclin-1*, have the potential to restore autophagic flux and prevent fibrotic processes [44,45]. Interventions targeting the Kelch-like ECH-associated protein 1 (KEAP1)-Nrf2 pathway are extremely successful in combating the ubiquitous oxidative stress in renal fibrosis. Activators such as bardoxolone methyl stabilize Nrf2, which increases the production of antioxidant proteins that minimize oxidative damage and reduce stress and fibrosis-causing inflammation [41].

The UPS, which degrades proteins, is critical to reducing proteotoxic stress. Dysregulation of this system contributes to fibrosis, and proteasome inhibitors reduce inflammation and decrease fibrotic signaling. These inhibitors stabilize critical proteins while inhibiting fibrogenic pathways, indicating a promising therapy approach [46]. Targeting NF-κB signaling can limit pro-inflammatory cytokine production and prevent fibrogenic pathways from activating, resulting in kidney fibrosis. Medications that control cytokines, like TNF-α and IL-6, can alleviate inflammation and fibrosis. Given the importance of mitochondrial dysfunction in fibrosis, medicines that improve mitophagy, a selective form of autophagy, are crucial. Agents that activate AMPK pathways minimize the buildup of ROS and damaged mitochondria, hence protecting renal cells from oxidative stress [47]. Combining treatment approaches has demonstrated synergistic advantages. Preclinical studies have shown that combining autophagy inducers with proteasome inhibitors can help alleviate proteotoxic stress, reduce inflammation, and prevent ECM deposition. These combination drugs address renal fibrosis in a holistic manner by addressing many interconnected pathways [48,49].

### 3.3. Therapeutic Advances Targeting the Ubiquitin–Proteasome System

#### 3.3.1. Proteasome Inhibitors

The UPS can be targeted by small-molecule inhibitors, with the most developed class of therapeutics being proteasome inhibitors. Bortezomib, a first-generation proteasome inhibitor, was the first medication approved by the U.S. Food and Drug Administration (FDA) to target the UPS. In hematologic malignancies such as multiple myeloma and mantle cell lymphoma, it has shown remarkable efficacy by blocking the chymotrypsin-like activity of the 26S proteasome. This leads to the accumulation of polyubiquitinated proteins and triggers apoptosis in malignant cells [50]. Researchers studying kidney disorders are interested in the potential of Bortezomib to regulate proteostasis and inflammatory signaling. In preclinical models of glomerular disorders, Bortezomib has shown promise in reducing inflammation and proteinuria. In the context of antibody-mediated renal rejection, Bortezomib increases transplant survival by reducing alloreactive plasma cells [51]. Bortezomib has been utilized as a treatment for AL amyloidosis by suppressing the manufacturing of amyloidogenic light chains, leading to an improvement in renal function. Noteworthy benefits in patients who used this medication have been reported [52]. Proteasome inhibitors have been investigated as potential treatments for various conditions, including lupus nephritis (LN) and idiopathic nephrotic syndrome, as well as antibody-mediated rejection and amyloidosis. In the case of lupus nephritis, these inhibitors have been shown to be effective in regulating autoreactive plasma cells and reducing the accumulation of immune complexes in the glomeruli [53]. Although Bortezomib has potential therapeutic uses, its utilization is constrained by unwanted side effects such as peripheral neuropathy and myelosuppression, which may occur as a result of off-target effects [54]. In order to develop more effective treatments for autoimmune conditions like LN and IgA nephropathy, research has concentrated on creating proteasome inhibitors that specifically target the immunoproteasome found predominantly in immune cells. These immunoproteasome inhibitors, such as KZR-616, have the ability to modify immune responses in a direct manner. By regulating the production of inflammatory cytokines and reducing glomerular damage, KZR-616 has the potential to offer a more viable solution for treating autoimmune disorders [55]. It is worth mentioning that the previously mentioned inhibitors, carfilzomib and ixazomib, are reportedly less toxic and more selective than other proteasome inhibitors. Although there is a lack of clinical trials directly testing their efficacy in CKD, preclinical research implies significant potential for these second-generation inhibitors in therapy. Additionally, delanzomib and marizomib are being evaluated for their ability to directly target immunoproteasomes, with the aim of reducing inflammation and systemic toxicity in kidney diseases [56]. The complex biochemical processes that underlie kidney damage in the conditions of DN and ischemia–reperfusion injury may be mitigated by pursuits aimed at inhibiting the proteasome. By reducing inflammation, oxidative stress, and fibrosis, inhibiting the proteasome may be able to meaningfully lower instances of kidney damage. One particular proteasome inhibitor, MG132, has been extensively studied and shown to be effective in mediating Akt-mediated inflammatory pathways. As a result, DN models have seen significant drops in renal inflammation and proteinuria [57]. Endothelin receptor antagonists and sodium–glucose cotransporter 2 (SGLT2) inhibitors are both effective in protecting the kidneys by decreasing fibrosis, oxidative stress, and inflammation [58,59]. When it comes to inhibiting fibrosis and restoring proteostasis, second-generation inhibitors are a superior option compared to first-generation inhibitors due to their higher degree of safety, lower toxicity, and increased selectivity. This is particularly relevant in the case of diseases that are associated with glomerular failure and tubulointerstitial fibrosis [60]. To gain a more comprehensive understanding of the impact of second-generation proteasome inhibitors on inflammation reduction, kidney function maintenance, and fibrosis prevention in CKD, further research is necessary. In particular, targeted clinical trials are needed to fully explore the potential of proteasome inhibitors in slowing the progression of CKD while also maintaining cellular viability (Table 2).

#### 3.3.2. DUB Inhibitors

The UPS can target deubiquitinating enzymes (DUBs) (Table 2), such as ubiquitin-specific proteases. These enzymes are critical to reversing ubiquitination, which regulates protein degradation and localization. Abnormal DUB activity has been associated with a variety of pathologies, including cancer and neurological disorders. In kidney diseases, malfunctioning DUBs can cause protein imbalance, inflammation, and fibrosis [63]. DUB inhibitors like b-AP15 target DUBs that interact with the proteasome, such as USP14 and UCHL5. When discussing polyubiquitinated substrates, it is important to note that inhibitors can prevent their processing, leading to a buildup of misfolded proteins and inhibiting proteases. In preclinical cancer models, the compound b-AP15 has been shown to increase oxidative stress and activate stress-related signaling pathways such as c-Jun N-terminal kinase/activator protein 1 (JNK/AP1), ultimately inducing apoptosis [64]. P5091, a selective DUB inhibitor, exhibits anti-inflammatory and antifibrotic characteristics because it modulates signaling pathways associated with kidney damage. P5091 inhibits the deubiquitination of critical substrates involved in cell cycle regulation and apoptosis, making it a promising treatment for CKD [65].

#### 3.3.3. KEAP1 Inhibitors

Other drugs involved in molecular targeting influence important pathways implicated in kidney disease progression, such as the KEAP1-Nrf2 axis and the UPS. In Table 2, there are inhibitors of KEAP1 listed, which may help in the treatment of kidney disease. These inhibitors stabilize and activate Nrf2, leading to the upregulation of antioxidant and cytoprotective genes that lower inflammation and oxidative stress. Studies conducted during the early phases indicated that patients with CKD who took these medications had lower renal fibrosis and higher glomerular filtration rates. Bardoxolone methyl, an Nrf2 activator, has demonstrated renoprotective effects in DN and Alport syndrome. However, there are concerns about fluid retention and heart failure that have limited its effectiveness [41]. Preclinical studies have shown that small-molecule inhibitors of the KEAP1-Nrf2 protein–protein interaction (PPI) have promising safety and therapeutic efficacy. One such inhibitor, CPUY192018, has been demonstrated to alleviate renal inflammation and oxidative stress in in vitro and in vivo models by promoting Nrf2 nuclear translocation and inhibiting NF-κB activation. This results in the protection of kidney cells from damage induced by lipopolysaccharide. The inhibitor has been shown to be potent and effective [72]. Another remarkable drug, UBE-1099, a non-covalent KEAP1 inhibitor, greatly restored kidney function in a mouse ischemia–reperfusion injury model. UBE-1099 minimizes tubular damage and macrophage infiltration without affecting body weight [66]. Recent structural modifications have also resulted in new, orally accessible inhibitors with strong action. In preclinical CKD models, for example, a fluorine-modified KEAP1 inhibitor activated antioxidant proteins such as heme oxygenase-1 in a dose-dependent manner [67]. AB38b, a synthetic α,β-unsaturated ketone, decreases oxidative stress and ECM production in DN by inhibiting KEAP1 and activating Nrf2. This drug successfully reduced fibrosis and oxidative indicators in diabetic mice kidneys, pointing to its potential as a nephroprotective medication [68].

## 4. Autophagy and Kidney Disease

The process of autophagy is essential for maintaining cellular balance and responding to various stressors, including calorie restriction, oxidative damage, and low oxygen levels (Table 1). Essentially, lysosomes engulf and break down damaged proteins, organelles, and other cytoplasmic components. Beclin-1 plays a role in autophagosome formation, while microtubule-associated protein light chain 3 (LC3) is crucial for their elongation and maturation [29].

Precise regulation of autophagy is crucial as improper activation or inhibition can disrupt the cellular balance and exacerbate kidney injury. Autophagy dysregulation has a significant impact on the functionality of key renal cell types, exacerbating cellular dysfunction and leading to kidney disease progression. When autophagy is interrupted, as in kidney diseases, the repercussions differ between renal cells, including podocytes, TECs, and glomerular endothelial cells. Podocytes, highly specialized epithelial cells that are essential for maintaining the GFB, rely heavily on autophagy to maintain precise cytoskeletal integrity and resist apoptosis when stressed. Impaired autophagy reduces the protein turnover, leading to cytoskeletal disintegration, foot process effacement, and proteinuria, all of which are hallmarks of glomerular sclerosis in diseases such as LN and DN [34]. Autophagy is essential for TECs to cope with cellular stress conditions resulting from situations such as ischemia–reperfusion and drug toxicity. When faced with cisplatin-induced nephrotoxicity, tubular autophagy promotes cellular defense against apoptosis by regulating the beclin-1 and LC3-II levels [61]. The malfunction of autophagy in TECs is associated with increased levels of lipotoxicity, oxidative stress, and mitochondrial damage, ultimately leading to the emergence of EMT, which is a forerunner of renal fibrosis [35]. Notably, AGEs, which are frequently elevated in DN, inhibit lysosomal activity and autophagic flow in TECs, resulting in the buildup of damaged organelles and proteins that worsen kidney injury [33]. In hypoxic conditions, glomerular endothelial cells utilize active autophagy as a protective mechanism for the glomerular barrier. The forkhead box O3 (FoxO3) transcription factor is responsible for activating autophagy, which induces the formation of autophagosomes. These protect endothelial cells from damage caused by oxidative stress resulting from urinary tract occlusion [69]. Autophagy plays a dual role in glomerular endothelial cells, with its effects depending on the specific situation. While basal autophagy is critical to maintaining cellular integrity and reducing oxidative damage under stressful conditions, excessive autophagy in autoimmune diseases such as LN can lead to inflammation and cellular dysfunction. Similarly, in DN, the failure of endothelial-specific autophagy exacerbates glomerulosclerosis by increasing capillary rarefication and compromising the glomerular filtration barrier [73].

Autophagy plays a dual role in kidney diseases, as it can either be protective or harmful depending on the situation. When AKI occurs, activating autophagy helps reduce apoptosis in TECs, as seen in cases such as ischemia–reperfusion injury and aristolochic acid nephropathy [74]. In contrast, excessive or dysregulated autophagy may contribute to cell death in chronic stress conditions such as persistent proteinuria or advanced DN, where the autophagic activity cannot keep up with the damage. High glucose levels might cause oxidative stress, which impairs the autophagic flow and leads to TEC apoptosis and kidney failure [75].

The dysfunction of autophagy in diabetic nephropathy affects both podocytes and TECs, leading to symptoms such as proteinuria, fibrosis, and progressive damage to the kidneys. Elevated levels of glucose inhibit autophagy by activating the mTOR signaling pathway, which contributes to increased oxidative stress and inflammation, resulting in podocyte loss and foot process deformation. Autophagic dysfunction in TECs has been linked to lysosomal membrane permeabilization caused by AGEs, which hinder lysosomal enzymatic activity and autophagic degradation. This irregularity can accelerate apoptosis and fibrosis, thereby exacerbating the progression of DN [76]. The link between autophagic failure and apoptosis is also visible in DN. In diabetic kidneys, inhibiting serum/glucocorticoid regulated kinase 1 (SGK1) in tubular cells was shown to increase autophagy while regulating EMT, fibrosis, and inflammation [77].

When utilizing DN models, rapamycin was observed to decrease both fibrosis and lipotoxicity by acting as an mTOR inhibitor and autophagy activator [31]. By activating the AMPK/mTOR pathway, it is possible to reduce damage to podocytes. Metformin, an AMPK activator, induces autophagy and can reduce lipotoxicity in renal TECs [78]. The activation of AMPK by emodin has been shown to stimulate autophagy in podocytes, resulting in reduced proteinuria and slowed renal fibrosis [79]. By nourishing podocytes with mangiferin, a natural activator of AMPK, it is possible to restore autophagic flux, decrease albuminuria, and stop the advancement of diabetic nephropathy through the activation of the AMPK-mTOR-ULK1 pathway [80]. To improve renal function in diabetic nephropathy, certain medications, including kaempferol, have renoprotective effects. These drugs induce autophagy in DN by activating the AMPK/mTOR pathways, reducing podocyte apoptosis, and expanding mesangial cells [62]. In DN models, hepatocyte growth factor (HGF) has the ability to improve podocyte autophagy by regulating the phosphoinositide 3-kinase (PI3K)/Akt–glycogen synthase kinase 3 beta (GSK3β)–transcription factor EB (TFEB) axis. Consequently, this results in decreased matrix enlargement and proteinuria [81].

The role of autophagy in LN is twofold, as podocytes exhibit heightened autophagic activity in order to safeguard against autoantibody-induced injury. To achieve this, mTORC1 is suppressed and oxidative stress resilience-promoting pathways are stimulated. Recent studies have indicated that boosting autophagy may have therapeutic benefits in LN. Rapamycin diminishes podocyte injury by increasing autophagic flux and maintaining the glomerular filtration barrier. Surprisingly, whereas basal autophagy is helpful, hyperactivation of autophagy in the LN might worsen inflammation by stimulating excessive immune complex buildup and endothelial cell damage. Therapeutic autophagy modulation, which balances activation without overactivation, is hence critical to effective LN management. Autophagic dysregulation can be targeted as a treatment for both DN and LN [30]. In order to treat diseases like LN with high levels of autophagic activity, autophagy inhibitors like chloroquine and hydroxychloroquine have been explored. These inhibitors work by reducing autophagosome–lysosome fusion and subsequently decreasing the amount of damage caused by autoantibodies [78].

To restore the protein balance in kidney diseases, gene therapy is a potentially effective strategy. Restoring autophagic flux in kidney cells may be facilitated by targeting genes linked to autophagy, such as *Beclin-1* and *Atg5*. In CKD patients, clustered regularly interspaced short palindromic repeat (CRISPR)-based strategies can help reduce protein aggregation, enhance UPS function, and modify E3 ubiquitin ligase activity. It has been demonstrated that CRISPRs that targets the KEAP1-Nrf2 axis shield renal tubular cells from oxidative damage [41].

Furthermore, nanosized drug delivery technologies are changing therapy choices by increasing the efficacy and safety of UPS and autophagy modulators. Bortezomib encapsulated in chitosan cross-linked polymeric nanoparticles showed enhanced cytotoxicity while demonstrating lower systemic toxicity in preclinical investigations [70]. Furthermore, gold nano-bipyramids coated with titania have been shown to lower autophagic flux while boosting the cytotoxicity of proteasome inhibitors, resulting in a synergistic effect that could be employed to treat kidney diseases [71].

## 5. Interplay Between UPS and Autophagy in Kidney Function

Despite their functional distinctions, recent research has revealed substantial crosstalk and cooperative relationships between the UPS and autophagy, which are essential for cellular survival and stress response. Both pathways interact in part because they use ubiquitin as a molecular marker. The proteasome or autophagy receptors like p62 (SQSTM1), neighbor of BRCA1 gene 1 protein (NBR1), and optineurin (OPTN) can destroy proteins that are polyubiquitin-tagged. The cargo is transported to autophagosomes through the interaction of these receptors with LC3 proteins, where it is subsequently destroyed. These receptors have the ability to recognize and transport ubiquitinated cargo to prevent further accumulation of damaged proteins. This dual-targeting capability improves protein quality regulation by allowing one system to compensate when the other is disrupted [82]. When Bortezomib inhibits the proteasome, this action results in heightened autophagy. This is due to the activation of the unfolded protein response and the inhibition of mTORC1. The transcription factor TFEB plays a key role in regulating this compensatory mechanism. It triggers lysosomal biogenesis and boosts the autophagic flow in response to increased proteotoxic stress [83].

The relationship between the UPS and autophagy is particularly significant in the podocytes and TECs, which are especially susceptible to proteotoxic stress because of their specific functions in filtration and reabsorption. For instance, in renal cells, p62 serves as an essential link for communication between pathways, facilitating the capture of ubiquitinated protein aggregates into autophagosomes for degradation. This function shields podocytes from cytoskeletal damage and inhibits TECs from undergoing EMT, a process that precedes fibrosis [73].

The simultaneous dysfunction of the UPS and autophagy exerts catastrophic effects on kidney function, leading to a cascade of consequences such as proteotoxic stress, mitochondrial dysfunction, inflammation, and fibrosis. These interconnected systems contribute to the development and progression of kidney diseases. When the proteasome fails, ubiquitinated proteins accumulate, potentially overwhelming the autophagy mechanism that eliminates these aggregates. This overload sets off a vicious cycle in which excess protein inhibits the autophagic flow, increasing proteotoxicity and threatening cellular homeostasis [84]. Abnormalities in podocytes can lead to foot process effacement, proteinuria, and, ultimately, glomerular sclerosis, which are key features of DN. Mitophagy, a form of autophagy that selectively removes damaged mitochondria, plays a crucial role in reducing oxidative stress. Inhibition of autophagy results in the accumulation of defective mitochondria, leading to increased levels of ROS and exacerbating cellular harm. This oxidative stress further impairs UPS function, perpetuating a cycle of organelle dysfunction and proteostasis collapse [85]. The breakdown of both pathways leads to the activation of pro-inflammatory signaling, such as NF-κB, resulting in the production of cytokines like IL-6 and TNF-α. Prolonged inflammation triggers TGF-β signaling, leading to the accumulation of ECM and, ultimately, renal scarring. Persistent fibrosis degrades the renal architecture, gradually affecting the filtration and reabsorption functions [86]. Targeting the interaction between the UPS and autophagy provides interesting therapeutic options. In preclinical CKD models, the combination of carfilzomib, a pharmacological proteasome inhibitor, with autophagy inducers like rapamycin has been utilized to enhance the management of proteotoxic stress and reduce kidney injury [87,88].

Combination therapies that simultaneously address the UPS and autophagy represent a viable approach for managing kidney diseases. Specifically, in preclinical CKD models, the synergistic use of proteasome inhibitors and autophagy inhibitors, such as hydroxychloroquine, has shown effectiveness in reducing protein aggregation and fibrosis [89,90]. These combined treatments provide a complex strategy for restoring proteostasis and reducing the progression of renal pathologies.

## 6. Conclusions

The UPS and autophagy are critical mechanisms that keep protein levels stable, protecting kidney cells from proteotoxic stress, oxidative damage, and inflammation. In kidney disorders, deregulation of these pathways upsets the equilibrium, leading to cellular malfunction, fibrosis, and apoptosis. Dysregulation of either system exacerbates kidney injury, and their combined dysfunction has a synergistic impact that accelerates disease development. In preliminary studies, the efficacy of treatment options targeting these pathways, such as proteasome inhibitors and autophagy modulators, appeared promising. Nonetheless, verifying these integrated approaches in human subjects via clinical trials is crucial to determining their safety and efficacy.

Insights into the proteostasis systems highlight the potential of biomarkers such as circular RNAs, ubiquitin ligases, and advanced glycation end-products to serve as early indicators of kidney injury, allowing for timely treatment. Pharmacological inhibitors of the UPS and autophagy modulators, such as rapamycin or metformin, provide chances to directly modify these pathways, potentially lowering inflammation, oxidative stress, and fibrosis in kidney disorders. In order to prevent the adverse effects (e.g., nephrotoxicity or autophagy hyperactivation) associated with use of these drugs, they need to be perfectly dosed and given to the right patients.

Novel therapies, including the combination drugs targeting the UPS and autophagy impaired, can provide additional protective effects against renal damage. Monitoring responses in the form of proteome and metabolomic data is one way to refine these approaches. In addition, the design of treatment technologies such as CRISPR-mediated gene therapies and nanocarrier-based drug delivery systems, carries potential for highly specific interventions, but this warrants additional research and clinical validation.

Despite the optimistic results, many research gaps must be addressed. Precision medicine approaches are critical to finding biomarkers that can predict individual responses to medications targeting autophagy and the UPS. The advent of omics technologies, like as proteomics and metabolomics [91], enables the identification of biomarkers that indicate UPS–autophagy interactions, potentially leading to personalized therapeutic regimens. Integrating these insights into routine clinical practice will necessitate a multidisciplinary approach, combining nephrology expertise with advancements in molecular medicine to enhance patient outcomes in terms of kidney disease management.

## Figures and Tables

**Table 1:** Key roles of the ubiquitin–proteasome system and autophagy in renal physiology and pathophysiology.

Aspect	Ubiquitin–Proteasome System	Autophagy	References
Primary function	Degradation of short-lived, misfolded proteins	Removal of long-lived proteins and organelles	[1,2]
Key molecular players	Ubiquitin, E1/E2/E3 enzymes, 26S proteasome	Beclin-1, LC3, p62/SQSTM1	[29,30]
Roles in renal physiology	Maintains podocyte cytoskeleton, regulates NF-κB signaling	Protects tubular cells from oxidative stress	[5,31]
Impact of dysregulation	Protein aggregation, inflammation, fibrosis	Mitochondrial dysfunction, epithelial injury	[32,33]
Associated diseases	Diabetic nephropathy, CKD, lupus nephritis	CKD, diabetic nephropathy, ischemic AKI	[34,35]

Abbreviations: AKI, acute kidney injury; CKD, chronic kidney disease; LC3, microtubule-associated protein 1A/1B light chain 3; NF-κB, nuclear factor kappa-light-chain-enhancer of activated B cells; SQSTM1, sequestosome 1.

**Table 2:** Therapeutic strategies targeting protein homeostasis in kidney diseases.

Category	Examples	Mechanism	References
Proteasome inhibitors	Bortezomib, carfilzomib, delanzomib and marizomib	Induce cell death by disrupting proteostasis	[50,54]
Autophagy modulators	Rapamycin, hydroxychloroquine	Activate or inhibit autophagy pathways	[31,61]
Gene therapy	Targeting beclin-1 or Atg5	Restores autophagic flux	[41,62]
DUB inhibitors	b-AP15, P5091	Block deubiquitination, inducing apoptosis and regulating inflammation	[63,64,65]
KEAP1 inhibitors	Bardoxolone methyl, CPUY192018, UBE-1099	Stabilize Nrf2 to upregulate antioxidant defenses and reduce oxidative stress	[42,66,67,68,69]
Nanocarriers	Bortezomib-loaded nanoparticles	Targeted delivery to enhance efficacy	[70,71]

## Data Availability

Not applicable.
